# Solitary abdominal wall hydatid cyst—a rare condition

**DOI:** 10.1093/jscr/rjac575

**Published:** 2022-12-09

**Authors:** Konstantinia Kofina, Nikolaos Papatheodorou, Michael Konomi, Ioannis Giannopoulos, Anastasios Valkanos, Michael Karanikas

**Affiliations:** 1st University Surgical Department, University Hospital of Alexandropoulis, Democritus University of Thrace, Alexandroupolis, Greece; 1st University Surgical Department, University Hospital of Alexandropoulis, Democritus University of Thrace, Alexandroupolis, Greece; 1st University Surgical Department, University Hospital of Alexandropoulis, Democritus University of Thrace, Alexandroupolis, Greece; 1st University Surgical Department, University Hospital of Alexandropoulis, Democritus University of Thrace, Alexandroupolis, Greece; 1st University Surgical Department, University Hospital of Alexandropoulis, Democritus University of Thrace, Alexandroupolis, Greece; 1st University Surgical Department, University Hospital of Alexandropoulis, Democritus University of Thrace, Alexandroupolis, Greece

## Abstract

Hydatid disease is a parasitic infection by a tapeworm of the genus Echinococcus that usually presents in the liver and lungs. Presentation of the disease as a solitary abdominal wall lesion, however, is a rare entity and only nine cases have been reported in the literature thus far. We present the case of a 53-year-old Caucasian female presenting with a mass located in the left flank that was proven to be a solitary hydatid cyst intraoperatively.

## INTRODUCTION

Hydatid cyst is common in countries of the temperate zones, where sheep, goats and cattle, its most common intermediate hosts, are raised. Most cases are, therefore, observed in the mediterranean region, northeastern Africa, Russia, South America and Australia [1]. The most usual locations of the disease are the liver lungs, but can also present in the spleen, bones, peritoneum, muscles, pancreas, brain and heart. Solitary abdominal wall hydatid disease, without involvement of liver or lungs is an extremely rare entity, with limited cases described as far [2]. This report outlines the case of a patient presenting with a solitary mass located in the left flank, which was proven to be a hydatid cyst intraoperatively.

## CASE REPORT

A 56-year-old Caucasian female patient from a rural area in northeastern Greece was referred to our Surgical Department for treatment of a solitary mass of the anterior abdominal wall in the left flank, which was first palpated 10 years before, with gradual enlargement. Her past medical and surgical history was free, and she did not mention any previous externa trauma in the left flank; however, she had a history of constant contact with cattle and dogs.

**Figure 1:**
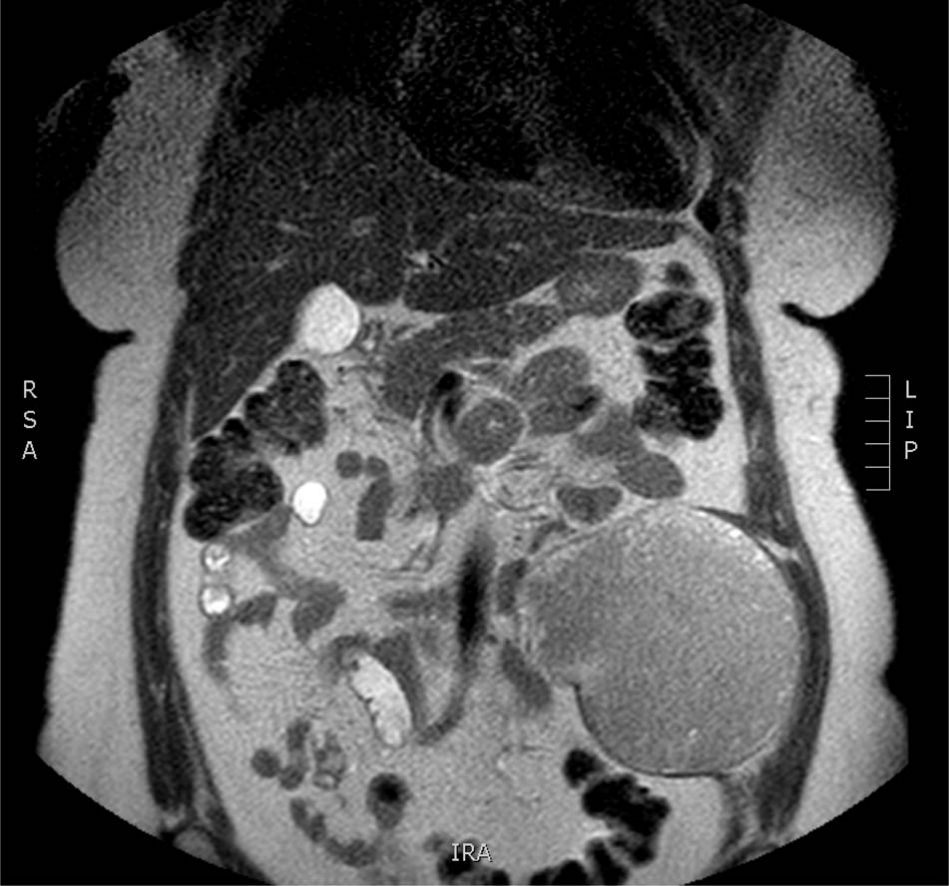
Preoperative MRI—axial plane. T1 weighted image. High sign density of the lesion.

**Figure 2:**
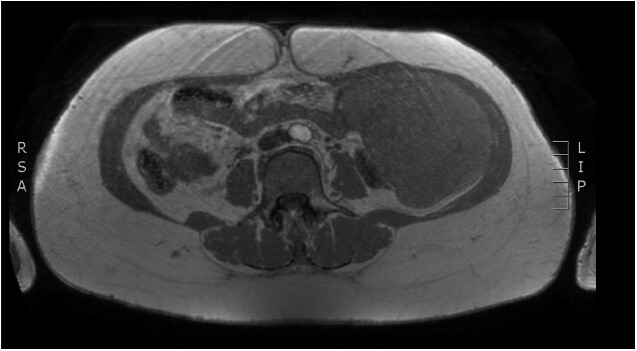
Preoperative MRI—saggital plane. T1 weighted image.

**Figure 3:**
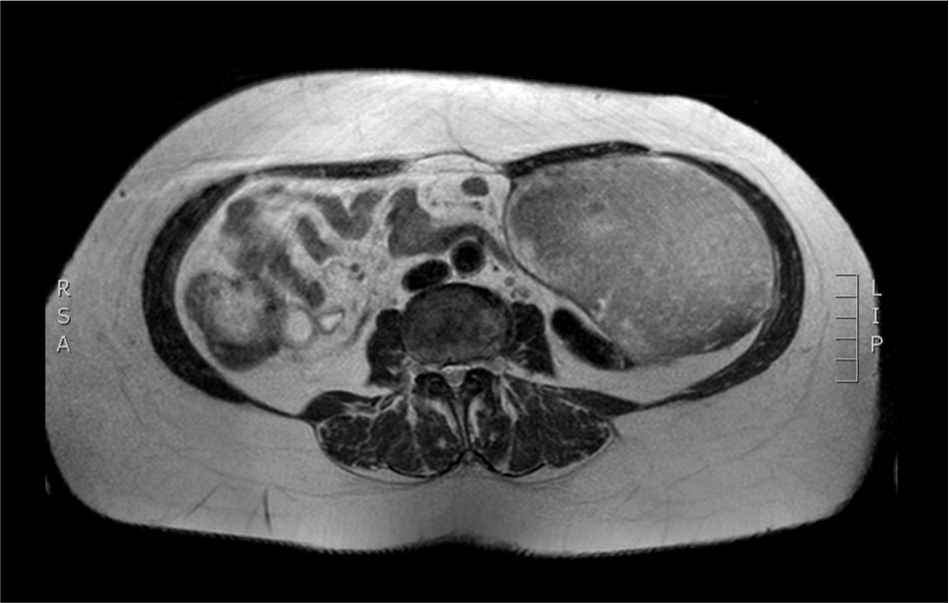
Preoperative MRI. T2 weighted image. Low sign density of the lesion.

Physical examination revealed a painless, mobile parietal mass on the left anterior abdominal wall, more prominent during cough, of ~13 cm in diameter, with smooth contour and without skin involvement. Laboratory examination including cancer marks showed results within normal limits; hydatid serology (IgG antibodies) was also negative. As ultrasonography results were inconclusive, further imaging with magnetic resonance imaging (MRI) revealed a sizable cystic mass measuring 10 × 13.4 × 11.7 cm, located in the left anterior abdominal quadrant, in the paraumbilical region, between the left internal oblique and the transversus oblique muscle, with projection to the inferior surface of the rectus abdominis. The mass was characterized as a thin-walled cystic lesion, with delayed uptake of intravenous contrast of the wall. High signal intensity was observed on T1-weighted images and low signal intensity on T2-weighted images, with high probability of neoplasia or bleeding (Figs 1–3). No other abdominal or pulmonary lesions were present.

**Figure 4:**
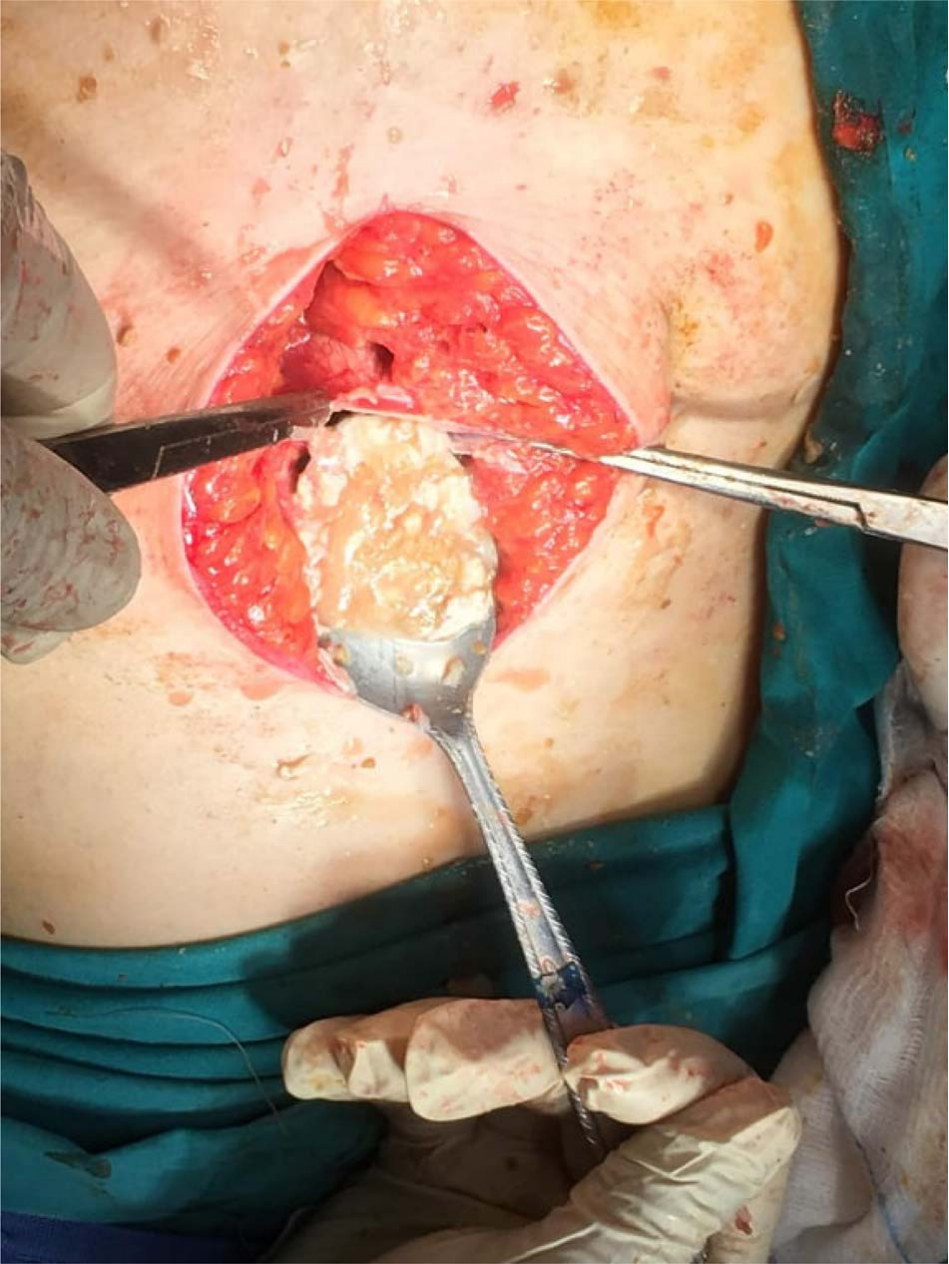
Intra-operative finding. Cyst content, indicating a hydatid cyst.

**Figure 5:**
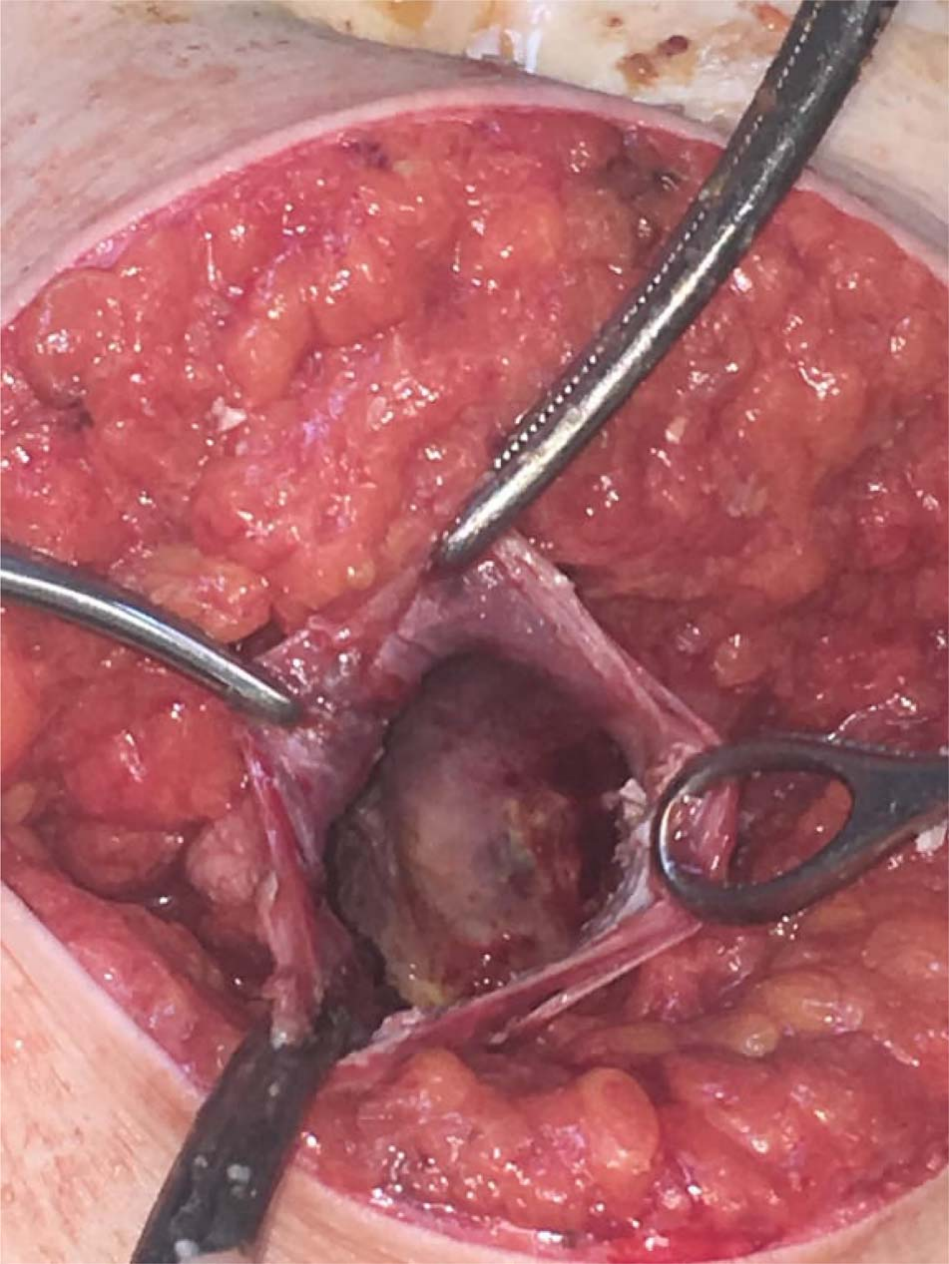
Total cyst drainage without spillage. Subsequent Pezzer tube placement through incision site.

Subsequently, surgical exploration was performed. A preperitoneal sizable cyst was revealed, which developed in the abdominal wall without the involvement of any abdominal viscera or wall peritoneum. The macroscopic appearance of its content suggested that the mass was indeed a hydatid cyst (Figs 4 and 5). Incision upon the cyst wall and evacuation of its content were performed, without spilling of the content. Hypertonic solution of sodium chloride (NaCl 15%) was then injected in the remaining cavity, followed by the placement of a Pezzer catheter.

The patient was discharged on the first postoperative day in good health condition and the Pezzer catheter was removed 10 days later. The histopathologic examination of the excised specimen confirmed the diagnosis of hydatid cyst and the patient was advised oral treatment with albendazole (400 mg twice a day, 3 days a week) for 3 months. Postoperative follow-up evaluation after 2 years has not detected any signs of recurrence.

## DISCUSSION

Hydatid disease is an endemic parasitic disease in Greece and, whereas more common in the liver and lungs, it can be located anywhere in the body. It is considered to be seen in the subcutaneous tissue without hepatic or lung involvement in only 2.3% of patients, whereas primary muscle infection accounts for 1–4% of reported cases [3]; the solitary localization in the abdominal wall is extremely uncommon [2–10], therefore its exact incidence is unknown.

Low prevalence of muscle and abdominal wall infection may be due to physical barriers to the hematogenous dissemination, probably caused by hepatic sinusoids and pulmonary capillaries, higher lactic acid concentration and contraction of the muscles. Possible pathways for the localization of the cyst in the abdominal wall musculature include direct parasite entry into the inferior vena cava and reflux implantation by Valsalva maneuver during daily activity, penetration from the intestines into the peritoneal space and subsequent invasion of the abdominal wall, or penetration into the abdominal lymphatics and subsequent localization in the abdominal wall musculature [2].

The diagnosis of hydatid disease relies on clinical findings, imaging techniques and serology. MRI findings, in particular, that are consistent with hydatid cyst include low-signal-intensity rim on T2-weighted images, which represents the collagen-rich pericyst, whereas cyst material presents hypointense, relative on T1- weighted images and hyperintense on T2- weighted images. Abscess, persistent hematoma, synovial cyst and neoplasia, such as sarcoma and liposarcoma, should be included in the differential diagnosis [4, 5]. Our patient lived in an endemic rural area; however, no previous history of hydatid disease was present, while serology and MRI findings were inconclusive, therefore preoperative diagnosis, could not be precise.

Treatment of cystic echinococcosis includes chemotherapy, cyst puncture and PAIR (percutaneous aspiration, injection of chemicals and reaspiration). However, surgical resection of the cyst remains the definitive standard of treatment [6, 7]. Drug treatment includes the use of anthelmintic drugs such as albendazole, mebendazole or praziquantel. In our case, as the cyst had been incised intra-operatively, careful drainage with subsequent anthelminthic therapy was opted as the best treatment that has also been mentioned in other studies [6], and no signs or recurrence have been present 2 years postoperatively.

In conclusion, hydatid cyst should be included in the differential diagnosis of all abdominal wall cystic masses, especially in endemic areas, even without previous history of the disease. Proper preoperative imaging is necessary in order to establish proper diagnosis and treatment.

## CONFLICT OF INTEREST STATEMENT

The authors declare that there are no conflicts of interest regarding the publication of this article.

## FUNDING

None.
